# Protamine-Induced Bradycardic Arrest in a Diabetic Patient

**DOI:** 10.7759/cureus.10955

**Published:** 2020-10-15

**Authors:** Brooke A McDonald, Kevin G Buda, Jeffrey R Hall, Michelle D Carlson, Robert Kempainen

**Affiliations:** 1 Internal Medicine, Hennepin County Medical Center, Minneapolis, USA; 2 Cardiology, Hennepin County Medical Center, Minneapolis, USA; 3 Pulmonary and Critical Care Medicine, Hennepin County Medical Center, Minneapolis, USA

**Keywords:** in hospital cardiac arrest, protamine sulfate, bradycardia, ventricular fibrillation (vf) storm

## Abstract

Protamine sulfate is a common reversal agent of systemic heparinization used during procedures. While the exact epidemiology of adverse events is unknown, prior allergic response to protamine-containing compounds or concomitant use of neutral protamine Hagedorn (NPH) insulin is associated with an increased risk of tachyarrhythmias and bradyarrhythmias. We present a case of a 68-year-old woman with no prior history of protamine sulfate intolerance that suffered bradycardic arrest following protamine infusion. Healthcare providers should recognize the potential for life-threatening tachyarrhythmias and bradyarrhythmias following protamine reversal, especially in diabetic patients at risk for autonomic dysfunction; medication and allergy review are encouraged prior to heparin reversal, especially in diabetic patients.

## Introduction

Protamine sulfate is a cationic polypeptide that binds to negatively charged unfractionated heparin and can be used to decrease the risk of bleeding during procedures in patients with systemic heparinization [1]. Though rare, severe side effects of protamine can occur, usually in patients with documented fish allergies or prior use of neutral protamine Hagedorn (NPH) insulin [1-3]. We present a case of a 68-year-old diabetic woman who experienced bradycardic arrest after receiving protamine sulfate. To the best of our knowledge, this is the first case report of protamine-induced bradycardic arrest in a patient without underlying medication allergies.

## Case presentation

A 68-year-old female with a past medical history of hypertension, peripheral artery disease, and type 2 diabetes complicated by peripheral neuropathy underwent a peripheral angiographic intervention on an occluded left femoral arterial stent, after which she received 20 mg of protamine. During the postoperative period, she experienced bradycardia and hypotension progressing to cardiac arrest. Cardiac monitoring at the time indicated no ventricular arrhythmias. Advanced cardiac life support (ACLS) was initiated and the patient received three rounds of epinephrine prior to transport to the emergency department (ED).

In the ED, initial evaluation demonstrated agonal cardiac activity on cardiac ultrasound. The patient received three more rounds of epinephrine, two amps of bicarbonate, and one gram of calcium chloride. The initial cardiac monitoring demonstrated pulseless electrical activity, which progressed to ventricular fibrillation. Defibrillation was performed, resulting in the return of spontaneous circulation (ROSC).

The patient was admitted to the medical intensive care unit. She was determined to be neurologically intact and targeted temperature management was not initiated. Medical history was notable for insulin NPH use. She had no prior allergic reaction to NPH, exposure to protamine sulfate, or history of food allergies.

**Figure 1:**
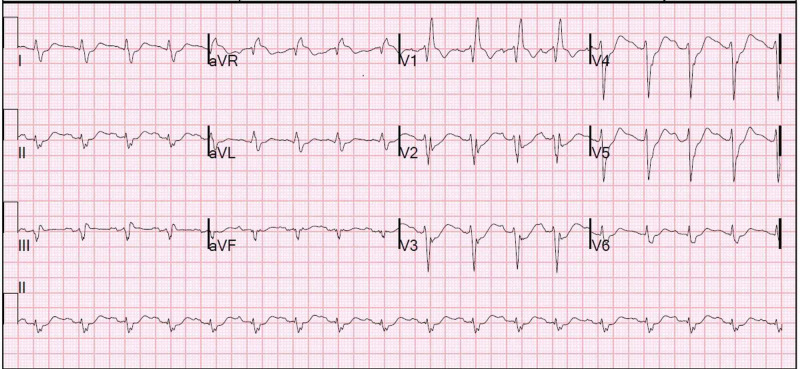
Initial ECG after ROSC showing sinus tachycardia with right bundle branch block, right axis deviation, and diffuse ST depression suggestive of subendocardial ischemia ROSC: return of spontaneous circulation.

Labs showed a rise and fall in troponin I with a peak value of 6.21 mcg/L (reference range ≤0.030 mcg/L). Initial electrocardiogram (ECG) after ROSC showed diffuse ST depression suggestive of subendocardial ischemia (Figure 1). Initial lab work demonstrated severe lactic acidosis, acute kidney injury, and elevated liver function tests suggestive of shock liver. Chest CT pulmonary angiogram was negative for pulmonary embolism. Coronary angiography showed mild coronary plaque without significant obstructive coronary artery disease, suggesting a type 2 myocardial infarction in the setting of an anaphylactic reaction to protamine. She completed 48 hours of unfractionated heparin for non-ST-segment elevation myocardial infarction (NSTEMI), continued dual antiplatelet therapy, and was placed on a high-dose statin and losartan upon resolution of her shock liver. NPH was discontinued indefinitely.

## Discussion

**Figure 2:**
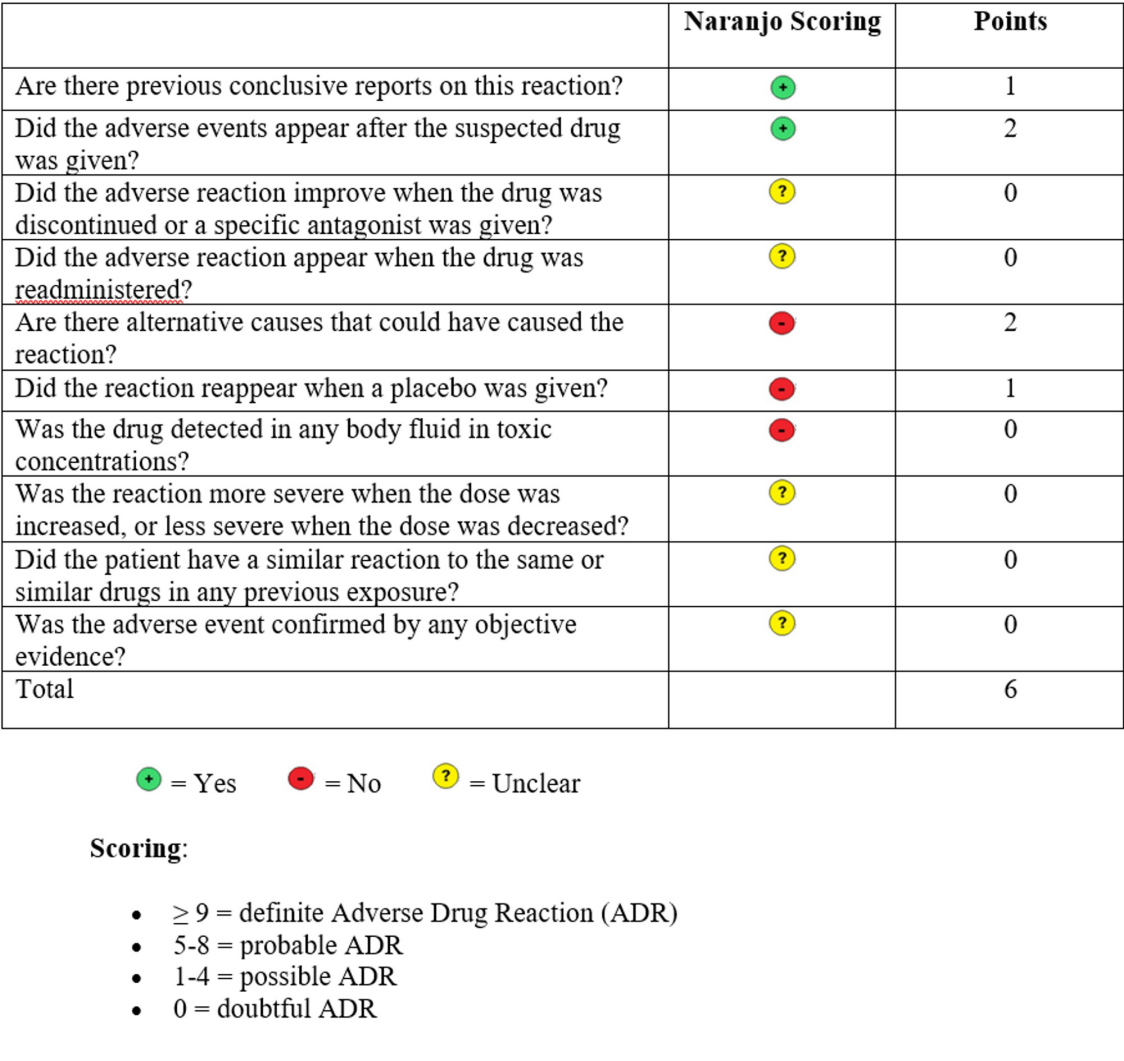
Likelihood that adverse drug reaction occurred due to the drug rather than the result of other factors, according to the Naranjo algorithm.

Ventricular fibrillation is a rare complication of protamine administration. Literature review yielded only one other case of bradycardic arrest after protamine administration, though this was in a patient with a documented history of allergies to both fish and NPH [4]. Adverse reactions associated with protamine sulfate include hypotension, pulmonary hypertension, bradycardia, anaphylaxis associated with type I hypersensitivity, and ventricular tachycardia or fibrillation [1,2]. Adverse effects have been associated with high doses of protamine sulfate, rapid administration, and prior exposure of protamine or protamine-containing compounds [5-6]. Prior protamine use - either via daily medications or invasive procedures requiring heparin reversal - is a mechanism for presensitization that can lead to an immunoglobulin E (IgE) mediated hypersensitivity reaction. In our patient, a true presensitization-mediated adverse drug reaction to protamine was likely (Figure 2).

Though the data on mechanisms of presensitization is both limited and heterogeneous, some studies report that individuals with fish allergies or those with prior exposure to protamine, such as diabetics using NPH insulin, can have up to a 50-fold increased risk of a major adverse reaction [3,7]. A meta-analysis of risk in NPH-using diabetic patients showed an odds ratio of 7.96 when comparing protamine associated adverse events to those without NPH exposure [7]. The odds ratio increased to 15.96 when comparing NPH surgical patients to non-NPH patients, suggesting that larger doses of protamine and prior exposure play a significant part in adverse outcomes in surgical patients [7].

## Conclusions

The pathophysiology of protamine-induced bradycardic arrest and the reason for its rarity in comparison to ventricular arrhythmias is unknown and an interesting area for further investigation, particularly given the known association between protamine and bradycardia. Further studies could also examine the role of anaphylaxis in long-term diabetic patients, as these patients may be at greater risk of protamine-induced arrhythmias due to unrecognized autonomic dysfunction. Though uncommon, the severity of potential reactions in patients receiving protamine sulfate for heparin reversal necessitates increased operator awareness, thorough chart review, and precise history-taking for prior allergies (including to NPH insulin and fish) and potential medication reactions. Healthcare providers should recognize the potential for bradyarrhythmias and tachyarrhythmias before protamine administration, particularly in diabetic patients.

## References

[1] Weiler JM, Freiman P, Sharath MD (1985). Serious adverse reactions to protamine sulfate: are alternatives needed?. J Allergy Clin Immunol.

[2] Leung LW, Gallagher MM, Evranos B, Bolten J, Madden BP, Wright BP, Kaba RA (2019). Cardiac arrest following protamine administration: a case series. EP Europace.

[3] Stewart WJ, McSweeney SM, Kellett MA, Faxon DP, Ryan TJ (1984). Increased risk of severe protamine reactions in NPH insulin-dependent diabetics undergoing cardiac catheterization. Circulation.

[4] Chu YQ, Cai LJ, Jiang DC, Jia D, Yan SY, Wang YQ (2010). Allergic shock and death associated with protamine administration in a diabetic patient. Clin Ther.

[5] SE Kimmel, MA Sekeres, JA Berlin, Ellison N, DiSesa V, Strom BL (1998). Risk factors for clinically important adverse events after protamine administration following cardiopulmonary bypass. J Am Coll Cardiol.

[6] Park KW (2004). Protamine and protamine reactions. Int Anesthesiol Clin.

[7] Vincent GM, Janowski M, Menlove R (1991). Protamine allergy reactions during cardiac catheterization and cardiac surgery: risk in patients taking protamine-insulin preparations. Cathet Cardiovasc Diagn.

